# Death of Sir James Y. Simpson, M. D., of Edinburgh

**Published:** 1870-06

**Authors:** 


### Death of Sir James Y. Simpson, M. D., of Edinburgh. Resolutions adopted by a meeting of American Physicians at Washington.

A meeting of such of the physicians of the United States as were then present at Washington, was held in the Army Medical Museum, May 10th, of which Dr. Thomas Miller was elected President, immediately upon the organizing of

*Dr. John P. Gray, Superintendent State Asylum, Utica; Prof. James P. White, of Buffalo; Dr. Wm. B. Gould, of Lockport; Dr. Milan Baker, of Warsaw, and Dr. Thomas D. Strong, of Westfield.

the meeting, Dr. H. R. Storer of Boston, in presenting resolutions, made the following remarks, as reported in the *Washington Chronicle:—*

Mr. Chairman and Brethren of the Profession:—It is with the deepest grief that I announce to you the decease, of which I have learned by cable dispatch from his son, of Prof. Sir James Y. Simpson, of Edinburgh. That our friend had himself prepared me by messages from his dying bed for his departure renders the loss none the easier to be borne. His very fortitude in his suffering, and his resignation to the Divine will, but revealed a more profound death and a lovelier beauty in his character, with which it was but the harder to part.

Dr. Simpson was so universally known to our profession throughout the world, as he was, indeed, to mankind at large, that I need enter into no detail concerning his history. He rose from the obscurity of a country village to be the favorite of his sovereign, the peer of the highest literary and scientific authorities, the cynosure of the medical and surgical intellect of this century. It was as if by magic, but his only talisman lay in the perfect bravery, persistency, sincerity, and simplicity of his life. Quick to perceive, he was equally apt in executing. He expended no unnecessary force, he begrudged no required effort. He was not merely the skilled accoucheur and the thoughtful, wise gynecologist; his suggestions regarding acupressure, now in daily practice, have placed him as a surgeon side by side with Ambrose Pare, while the revolution achieved by him as to the fundamental idea of hospitalism entitles him to the glorious appellation of having been the modern father of medicine, a second Hippocrates.

There are those present who, from personal acquaintance with the facts, know that these claims are in no sense forced or overstated. At less than sixty he had accomplished all that has been said, and yet, still in the fresh prime of his life, we hoped for even riper fruits from his vast experience. But it was not so ordained. His last professional work, sent to us from his death-bed, was for the vindication of the honor of one of our own countrymen, whose memory, thus redeemed, will always be embalmed in our hearts conjoined with that of Simpson. His reply to the second letter of Dr. Jacob Bigelow concerning the history of Practical Anæsthesia, communicated to the Gynæcological Society, of Boston, and received through it by you at the session of the American Medical Association just ended, went far to influence your decision as to the person to whom the honor of that glorious discovery, the most beneficent ever made since the foundation of the world, was really due. By an unanimous vote upon the last day of the session, and in pursuance of the recommendation of the Section of Practical Medicine, by whom the evidence adduced had been carefnlly scrutinized, you pronounced in favor of the late Dr. Horace Wells, of Hartford, Conn. Upon the evening of the same day—his earthly labors thus beautifully ended—the spirit of Dr. Simpson took its flight.

There would be no place in this country so fitting for these few words of eulogy, this poor utterance of our common gratitude, as the national capital; and this Army Medical Museum, filled as it is with the results of achievements, formerly impossible, is in itself his fitting monument. Coming together from the uttermost parts of the continent, we go hence to homes where his name is everywhere a household word, never to be forgotten so long as the primal curse, from which, through God's great grace, he took the sting, shall lie upon suffering woman. The nations shall rise up indeed to call him blessed, and blessed he is, if to have faithfully worked through his whole life in the Lord's vineyard, in season and out of season, cheerfully doing that which was given him to do, bearing up under the heaviest domestic afflictions, not resenting unkindly the sarcasms and ingratitute of petty men, assuming withal for the dear Saviour's sake. His heavy cross, the jeers of sceptics and the taunts of those who daily crucify him anew—if to have lived thus is to have gained entrance into the joy of the Lord, blessed he is indeed.

Of my own personal bereavement I have now nought to speak. There may be those present, however, who will remember my language of fifteen years ago, in the preface to the American edition of those "Memoirs and Contribubutions," to edit which, in conjunction with Dr. Priestley, now of London, it had been my great privilege to be selected. "Treating me as his son, I had learned to love him as a parent." As such, indeed, I have found the tie that is now broken. There are sorrows that can not express themselves in words. I can only offer you the following resolutions. They will be found but feebly to convey what I know is in all your hearts:

Whereas it is an instinctive and very natural desire among men to lament with those who are in affliction, and to mourn with those who weep; and whereas it has pleased the Giver of both mortal and eternal life to call unto Himself His good and faithful servant, known upon earth as Dr. James Y. Simpson, of Edinburgh: therefore,

*Resolved.* That in Dr. Simpson American physicians recognize not merely an eminent and learned Scotch practitioner, but a philanthropist whose love encircled the world; a discoverer who sought and found for suffering humanity, in its sorest need, a foretaste of the peace of Heaven, and a devoted disciple of the only true physician, our Saviour, Jesus Christ.

*Resolved.* That in acknowledging, for ourselves and our brethren, the excellence of him who has gone, and in thus honoring his memory, we would tender to the members of his family in their sorrow our respectful sympathy.

*Resolved.* That a copy of these resolutions be sent to the widow of Sir James Simpson, and to the British Minister resident at Washington, with the request of the latter that they be transmitted by him to the several English medical journals as a mark of the esteem felt in this country for the deceased.

Dr. Geo. A. Otis, U. S. A., in his remarks, said that he wished that the incalculable indebtedness of military surgeons to that wisdom and courage which was manifested in the discovery of chloroform, might be fittingly appreciated and due honor paid to one, who has conferred upon army surgeons, the greatest boon they have received since Ambrose Pare discovered the ligature, and who deserves as well as Pare, to be called a man-lo[illegible]ng, king-honored, God-serving physician. In our late struggle, chloroform [illegible]as administered in more than one hundred and twenty thousand cases, and he was unable to learn of more than eight cases in which a fatal result was fairly traceable to its use.

Dr. Geo. Clymer, U. S. N., remarked feeling[illegible]y upon the great obligations the medical staff of the navy, sustained towards Prof. Simpson.

Dr. C. C. Cox, said that:—Distinguished as Sir James Simpson was in the *specialities* to which so much of his life was devoted, he can hardly be said to have been *homo unius libri*, since he acquired distinction in physics, belle-letters, and theology. It may be said that he was the Mæcenas of the ancient and classic city of Edinburgh. His house was ever open to men of letters, and his breakfast table especially, (as I have good reason to know,) was never without representatives of the various departments of useful knowledge. It was on these occasions that his genial character, unobtrusive modesty, and vast attainments were most conspicuously manifested.

To Americans he was especially kind and courteous. I remember most gratefully my own cordial reception at his hands. It was my good fortune to be under his roof, to ride with him on his daily professional rounds, and to visit his modest infirmary, so familiar to my friend, Dr. Storer, and I can truly say that my intercourse with that great and good man forms one of the most agreeable recollections of my foreign sojourn.

Gen. R. Mussey, then in behalf of the other learned professions and the public, gave an impressive eulogy of the life and entertainments of Dr. Simpson. After remarks by others, upon motion by Drs. Garnett and Young, it was voted that a copy of the proceedings of the meeting be given to the daily papers, also to the Secretary of the American Medical Association. Dr. Hull then moved that the resolutions offered by Dr. Storer and seconded by Dr. Johnston, be signed by the members of the meeting, which was adopted. The meeting comprised a large number of the principal physicians of the District of Columbia, also many from distant parts of the country.

